# Moderate or greater daily coffee consumption is associated with lower incidence of metabolic syndrome in Taiwanese militaries: results from the CHIEF cohort study

**DOI:** 10.3389/fnut.2023.1321916

**Published:** 2023-12-14

**Authors:** Kun-Zhe Tsai, Wei-Chun Huang, Xuemei Sui, Carl J. Lavie, Gen-Min Lin

**Affiliations:** ^1^Department of Stomatology of Periodontology, Mackay Memorial Hospital, Taipei, Taiwan; ^2^Department of Medicine, Hualien Armed Forces General Hospital, Hualien City, Taiwan; ^3^Department of Periodontology, School of Dentistry, National Defense Medical Center and Tri-Service General Hospital, Taipei, Taiwan; ^4^Department of Critical Care Medicine, Kaohsiung Veterans General Hospital, Kaohsiung, Taiwan; ^5^College of Medicine, National Yang Ming Chiao Tung University, Taipei, Taiwan; ^6^Arnold School of Public Health, University of South Carolina, Columbia, SC, United States; ^7^Ochsner Clinical School, John Ochsner Heart and Vascular Institute, The University of Queensland School of Medicine, New Orleans, LA, United States; ^8^Department of Medicine, National Defense Medical Center, Tri-Service General Hospital, Taipei, Taiwan

**Keywords:** coffee intake, cohort study, metabolic syndrome, young adults, military health

## Abstract

**Background:**

Daily moderate coffee intake was found with a lower risk of specific metabolic abnormalities, e.g., hypertension and hyperglycemia, while the association of coffee intake and incident metabolic syndrome (MetS) has not been clarified in prior studies, particularly in young adults.

**Methods:**

A total of 2,890 military personnel, aged 18–39 years, free of MetS were followed for incident MetS from baseline (2014) until the end of 2020 in Taiwan. Daily coffee amount consumed was grouped to those ≥3 cups or 600 mL (moderate or more amount) and those without. Incidence of MetS was identified in annual health examinations. MetS was diagnosed on the basis of the guideline of the International Diabetes Federation. Multivariable Cox regression model with adjustments for sex, age, body mass index, physical activity and substance use status at baseline was performed to determine the association.

**Results:**

At baseline, there were 145 subjects with daily coffee intake ≥3 cups or 600 mL (5.0%) in the overall cohort. During a mean follow-up of 6.0 years, 673 incident MetS (23.3%) were found. As compared to those consuming less coffee or none, those consuming daily coffee ≥3 cups had a lower risk of MetS [hazard ratio (HR): 0.69 (95% confidence interval: 0.48, 0.99)].

**Conclusion:**

This study suggests that adhering to the guideline recommended moderate or greater daily coffee consumption for promoting health, may confer advantages in preventing the development of MetS among young adults.

## Introduction

Metabolic syndrome (MetS) represents a combination of cardiovascular risk factors, including insulin resistance, dyslipidemia, obesity, and hypertension. Despite variations in its definition, MetS is estimated to affect approximately a quarter of the global population and has the largest global burden of all non-communicable diseases (1, 2). Over the past three decades, the prevalence of MetS in the United States has surged by nearly 30% (3). European studies have reported a prevalence of 23.9% in men and 24.6% in women (4). In Asia, nearly one-fifth of the adult population or more has suffered from MetS, with a steady increase in prevalence over time (5). This heightened prevalence is concerning because MetS is closely linked to an increased risk of developing cardiovascular diseases (CVD) and experiencing all-cause mortality (4). Individuals with MetS face a twofold increased risk of CVD over 5–10 years and a fivefold or greater risk of developing type 2 diabetes over their lifetime (1). Therefore, it is crucial to prioritize preventive measures (1, 6).

Coffee is a beverage rich in bioactive compounds such as caffeine, melanoids, chlorogenic acid, and polyphenols, known for the potential health benefits, e.g., antioxidative, anti-inflammatory, and anti-carcinogenic effects (7). Population-based studies have suggested that consuming two to four cups (approximately 473–946 mL or 16–32 oz.) of typical hot-brewed or reconstituted instant coffee daily is associated with a reduced risk of various illnesses, including diabetes, CVD, and specific metabolic abnormalities like obesity and hypertension (8, 9). However, previous research on the relationship between coffee consumption and the presence of MetS showed conflicting results (6, 9–16). One significant limitation in many of these studies has been the absence of information about physical activity (PA), a crucial factor that can profoundly influence metabolic health. Therefore, it is essential to explore the complex interplay between coffee intake, PA levels, and the risk of MetS to gain a comprehensive understanding of their potential associations and interactions.

## Methods

### Study population

The cohort study enrolled 4,080 military personnel, aged 18–50 years, as part of the cardiorespiratory fitness and health in Eastern armed forces (CHIEF) study in Taiwan of the Republic of China in 2014 (17, 18). Each participant underwent annual health examinations, which included measurements of anthropometrics, hemodynamics, and blood biomarkers. In addition, participants reported their substance use status, i.e., alcohol consumption, betel nut chewing, and tobacco smoking, classified to active and former/never users, as well as their moderate-intensity PA levels evaluated by leisure-time running (<150, 150–299, and ≥300 min/week) over the past 6 months. This information was collected through a self-administrated questionnaire of the health report at the Hualien Armed Forces General Hospital at the baseline assessment in 2014 (19–21). This study adhered to the principles outlined in the Declaration of Helsinki. Furthermore, the study’s protocol was reviewed and approved by the Institutional Review Board (IRB) of Mennonite Christian Hospital in Hualien City, Taiwan, with certificate number 16-05-008. Written informed consent was obtained from all participants.

### Coffee consumption

Participants’ coffee consumption habits were assessed through a self-reported questionnaire, simultaneously with the report of substance use and PA levels. This questionnaire inquired about participants’ coffee consumption over the past 6 months and specifically focused on whether, on average, they consumed on average or more than 3 cups or 600 mL per day, approximately the guideline recommended moderate coffee amounts for promoting health (8, 9).

### Annual military health examinations, 2014–2020

The anthropometric measurements, including waist circumference (WC), body height, and weight, were taken once with the subjects in a standing position. Body mass index (BMI) was subsequently calculated as the ratio of body weight (in kilograms) to the square of body height (in square meters). Measurement of WC was performed by an experienced technician to place the tape midway between the top of the subject’s hip bone and the bottom of ribs in line with the belly button, and wrap it around the waist loose enough to fit one finger inside the tape when the subject breathed out smoothly.

Resting blood pressure (BP) was measured once for each participant while in a seated position utilizing an oscillometric method from an automatic BP device (FT201 Parama-Tech Co., Ltd., Fukuoka, Japan) (22–24). In cases where the initial systolic/diastolic BP reading was equal to or exceeded 130/80 mmHg, a second measurement was performed after a 15-min rest period. The final BP level reported was the average of the initial and second BP measurements.

Concentrations of serum total cholesterol, low-density lipoprotein cholesterol (LDL-C) and high-density lipoprotein cholesterol (HDL-C), triglycerides and fasting plasma glucose (FPG) were determined from overnight 12-h fasting blood samples collected from each subject. These measurements were analyzed using an automated analyzer (Olympus AU640, Kobe, Japan) (25).

### Definition of MetS

As per the International Diabetes Federation’s criteria tailored for the Chinese population (26), MetS was diagnosed as having three or more of the following clinical features: (1) WC ≥90 cm for men and WC ≥80 cm for women; (2) HDL-C < 40 mg/dL for men and <50 mg/dL for women; (3) Serum triglycerides ≥150 mg/dL or the use of lipid-lowering medications; (4) FPG ≥100 mg/dL or the use of anti-diabetic medications; (5) Systolic BP ≥130 mmHg, or diastolic BP ≥85 mmHg, or the use of antihypertensive therapy (27).

### Statistical analysis

The military young cohort’s baseline characteristics were presented using mean ± standard deviation (SD) for continuous factors and numbers (percentages) for categorical factors. The follow-up for each subject began in 2014 (baseline) and lasted until the first occurrence of MetS event, loss to follow-up, or the end of the follow-up, which was by December 31, 2020. We employed Kaplan–Meier Curve to analyze the survival rate (free of incident MetS) between two groups based on their coffee consumption status, and a difference was assessed using the log-rank test. Multivariable Cox hazards regression analysis was utilized to investigate the hazard ratios (HR) and 95% confidence intervals (CI) between coffee intake status and the incidence of MetS with simultaneously adjustments for baseline age, sex, BMI, alcohol consumption status, betel nut chewing status, tobacco smoking status, and PA levels. Subgroup analyses that stratified by sex (men and women), age (≥30 and <30 years), BMI (≥25.0 and <25.0 kg/m^2^), PA activity (≥300 and <300 min/week) and alcohol intake status (active and nonactive) were conducted, and formal testing for interaction was performed. In addition, for the five MetS components, LDL-C and total cholesterol, analysis of covariance (ANCOVA) with adjustments for the relevant metabolic marker level and the priorly mentioned covariates was used to determine whether the difference between the Year 5 and baseline levels of each metabolic marker of the two groups was present. Statistical significance was defined as a *p*-value < 0.05. All statistical analyses were carried out using SPSS version 26.0 for Windows, developed by IBM Corp. in Armonk, NY, United States.

## Results

In this study, participants with baseline MetS (*N* = 457), those aged 40 years or older at baseline (*N* = 58), and those who were transferred out of military bases in Eastern Taiwan and subsequently lost to follow-up (*N* = 675) were excluded. This results in a final population of 2,890 subjects for the analysis.

The baseline characteristics of the study cohort are presented in Table 1. In the final analytical sample, participants with daily coffee intake ≥3 cups or 600 mL had an older mean age and a higher prevalence of active betel nut chewing and alcohol intake, whereas the difference in PA levels was statistically marginal (*p* = 0.09).

**Table 1:** Baseline characteristics of military participants free of baseline metabolic syndrome.

	Daily coffee intake <3 cups or 600 mL (*N* = 2,745)	Daily coffee intake ≥3 cups or 600 mL (*N* = 145)	*p-*value
Age, years	28.29 ± 5.79	30.10 ± 5.59	<0.001
Male sex, %	2,453 (89.4)	128 (88.3)	0.68
Alcohol intake, %	1,078 (39.3)	83 (57.2)	<0.001
Betel nut chewing, %	256 (9.3)	23 (15.9)	0.009
Cigarette smoking, %	936 (34.1)	55 (37.9)	0.34
**PA levels, %**			
<150 min/week	593 (21.6)	34 (23.4)	0.09
150–299 min/week	1,039 (37.9)	65 (44.8)	
≥300 min/week	1,113 (40.5)	46 (31.7)	
BMI, kg/m^2^	24.27 ± 3.01	24.33 ± 2.83	0.79
Waist circumference, cm	81.43 ± 8.06	81.60 ± 8.00	0.81
Systolic blood pressure, mmHg	115.94 ± 12.93	114.93 ± 12.35	0.36
Diastolic blood pressure, mmHg	69.29 ± 9.58	69.51 ± 10.85	0.79
Serum total cholesterol, mg/dL	171.79 ± 32.49	175.64 ± 33.84	0.16
Serum low-density lipoprotein, mg/dL	103.70 ± 28.94	106.23 ± 29.26	0.30
Serum high-density lipoprotein, mg/dL	49.90 ± 9.90	50.35 ± 12.01	0.60
Serum triglycerides, mg/dL	96.91 ± 58.98	102.94 ± 53.23	0.20
Fasting plasma glucose, mg/dL	91.96 ± 9.74	92.60 ± 9.57	0.44

Continuous variables are presented as mean ± standard deviation (SD), and categorical variables as N (%).

BMI, body mass index; PA, physical activity.

**Figure 1:**
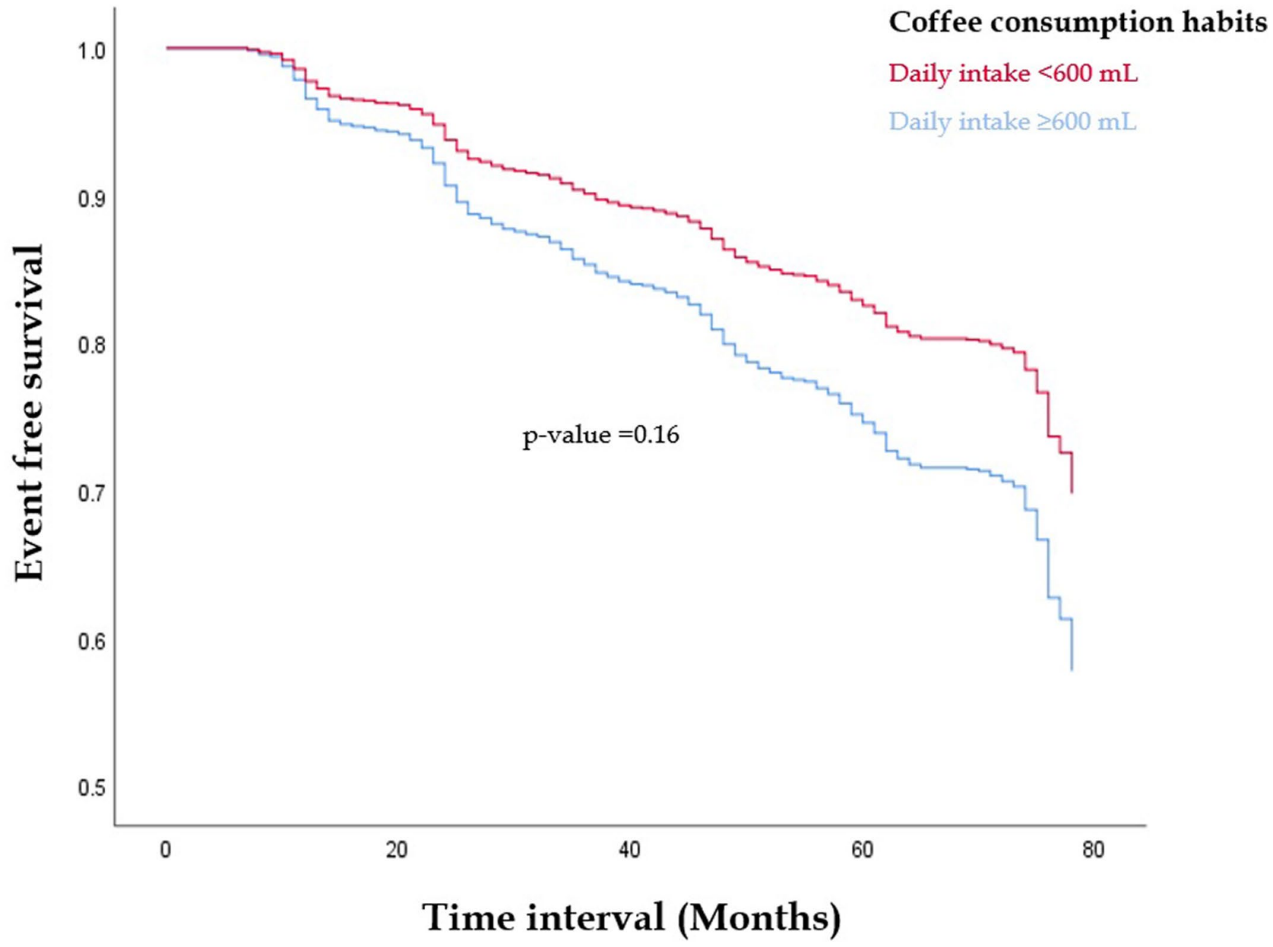
The Kaplan–Meier survival analysis for free of metabolic syndrome events in those with daily coffee consumption ≥3 cups or 600 mL compared to their counterparts consumed none or less daily coffee amount (*p* = 0.16).

Figure 1 reveals the result of the Kaplan–Meier analysis. Participants with daily coffee consumption ≥3 cups or 600 mL showed a lower incidence of MetS event as compared to their counterparts consumed none or less daily coffee amount. Nevertheless, this disparity was not statistically significant (*p* = 0.16).

Table 2 shows the multivariable Cox proportional hazards regression analysis results. In the crude Model, the inverse association between coffee intake status and the risk of MetS was null (HR: 0.77, *p* = 0.16) which was consistent with the finding of Kaplan–Meier analysis. On the contrary, in the multivariable Model, as compared to participants with a daily coffee intake <3 cups or 600 mL, those who consumed daily coffee intake ≥3 cups or 600 mL exhibited a lower risk of developing new-onset MetS [HR: 0.69 (95% CI: 0.48, 0.99), *p* = 0.04]. The results of subgroup analyses are shown in Supplementary Table S1. We found that there were no significant differences in subgroup analyses according to age, sex, BMI, alcohol intake and PA level status.

**Table 2:** Multivariable cox regression analysis for incidence of metabolic syndrome with coffee intake status.

Coffee intake	Crude model			Multivariable model		
	HR	95% CI	*p*-value	HR	95% CI	*p*-value
<3 cups or 600 mL/day	1.00					
≥3 cups or 600 mg/day	0.77	0.54–1.11	0.16	0.69	0.48–0.99	0.04

Data are shown as hazard ratio (HR) and 95% confidence interval (CI).

Multivariable Model: with adjustments for age, sex, body mass index, alcohol intake, betel nut chewing, cigarette smoking and physical activity.

Table 3 reveals the results of ANCOVA for the 5-year interval change in each metabolic biomarker level of the two groups. As compared to those with daily coffee intake <3 cups or 600 mL, the increased levels of WC, FPG, BP and serum triglycerides in those with daily coffee intake ≥3 cups or 600 mL were lower, in which a significant difference was merely present for WC (*p* = 0.02). In contrast, the 5-year interval increases in total cholesterol and LDL-C levels were greater in participants with daily coffee intake ≥3 cups or 600 mL, in which a significant difference was present for LDL-C (*p* = 0.01).

**Table 3:** Changes in the metabolic biomarkers levels between year 5 and baseline (year 0) in participants with moderate or more daily coffee intake and those without.

Differences (Year 5 – Year 0)	Daily coffee intake <3 cups or 600 mL	Daily coffee intake ≥3 cups or 600 mL	*p*-value
Δ Waist circumference, cm	3.48 ± 8.55	3.21 ± 9.86	0.02
Δ Systolic blood pressure, mmHg	−0.27 ± 12.56	−1.20 ± 10.90	0.42
Δ Total cholesterol, mg/dL	9.60 ± 22.89	11.02 ± 22.40	0.12
Δ Low density lipoprotein, mg/dL	3.43 ± 19.29	5.92 ± 19.66	0.01
Δ High density lipoprotein, mg/dL	−2.50 ± 7.05	−2.72 ± 8.04	0.70
Δ Serum triglycerides, mg/dL	27.81 ± 86.92	26.90 ± 60.63	0.61
Δ Fasting glucose, mg/dL	3.25 ± 11.83	1.20 ± 11.32	0.13

Analysis of covariance (ANCOVA) was used to compare the 5-year interval change in each metabolic component level between two groups.

Adjustments for baseline age, sex, body mass index, alcohol intake, betel nut chewing, cigarette smoking, physical activity and relevant metabolic biomarker level.

## Discussion

This study suggests that moderate or greater daily coffee consumption based on the guideline recommendation may be beneficial in preventing new-onset MetS among young adults, regardless of a greater increase in total cholesterol and LDL-C concentrations. In addition, this longitudinal cohort study was the first report providing substantial evidence for the effect of coffee consumption on primary prevention of the development of objectively diagnosed MetS with simultaneously adjustment for PA levels at baseline in young adults.

While previous prospective cohort studies examining the preventive effects of coffee intake on MetS were limited, their findings were inconsistent (6, 10–13). Most of the previous cohort studies failed to establish a significant connection between coffee consumption and the development of MetS (10–13). These prior investigations exhibited heterogeneity in various aspects, e.g., the participants’ average baseline age (ranging from 20 to 65 years), the duration of follow-up (spanning from 6 to 22 years), the methods used to assess coffee consumption, and without adjusting for baseline crucial confounding factor such as PA levels. Nevertheless, a recent study involving 10,253 participants with a 6-year follow-up period suggests that daily coffee consumption 1–4 cups may be associated with a reduced risk of new-onset MetS in young adults [odds ratio: 0.71 (95% CI: 0.50, 0.99)] (6). However, the finding was restricted by the diagnosis of MetS depending on the subjective self-report questionnaire. In the context of cross-sectional study, the association between coffee consumption and MetS remains contentious (9, 14–16). The disparities might be due to types and quantities of coffee consumed, and by PA levels not consideration, which are a significant modifier of metabolic health (14).

Over the past decade, a growing body of research has found that caffeine and its methylxanthine metabolites could affect oxidative stress and inflammation pathways (28). Chronic caffeine administration could suppress reactive oxygen species (ROS) and nuclear factor kappa-light-chain-enhancer of activated B cells (NF-κB) pathway (28). Notably, caffeine’s effect on inflammation may vary with dosage; low doses may exacerbate acute inflammation, while higher doses can mitigate it (29, 30). Furthermore, caffeine and its metabolites could affect lipid and glucose metabolism through a reaction on enzyme activity (31, 32). Specifically, caffeine can inhibit phosphodiesterases, leading to increased cyclic adenosine monophosphate (cAMP) levels, which promote lipolysis processes (33, 34). In addition, caffeine regulates fat metabolism through the sympathetic nervous system, promoting the secretion of catecholamines that activates β-adrenergic receptors and downstream pathways for lipid metabolism (34, 35).

The phenolic compounds in coffee extract serve as a defense mechanism for vital cellular components, safeguarding them from harm caused by reactive free radicals. Their effectiveness lies in their capacity to activate antioxidant enzymes, mitigating oxidative stress and inflammation (36). In addition, the phenolic metabolites can act as inhibitors or isolators of ROS while transferring electrons to free radicals, thereby promoting metabolic health (37). In this case, polyphenols in coffee could modulate metabolic pathways, contributing to the regulation of MetS characteristics (e.g., reduced serum triglycerides and FPG). On the contrary, the findings of this study also found moderate or more coffee intake may increase LDL-C and total cholesterol concentrations. Although the previous findings for the effect of coffee consumption on lipids concentrations were inconsistent, some meta-analyses revealed a dose response association of coffee intake amounts with total cholesterol and LDL-C concentrations (38, 39). Diterpenes in coffee and unfiltered coffee were found to raise these atherogenic cholesterols, particularly in individuals with baseline hyperlipidemia (39, 40). Since total cholesterol and LDL-C were not a component of MetS, the incidence of MetS with moderate or more coffee intake in this study was not affected.

### Strengths and limitations

This study possesses certain strengths and limitations. On the positive side, the findings benefited from the detailed baseline characteristics of participants, allowing for the adjustment for potential confounding factors, particularly PA levels. Additionally, military personnel share similarities in their environment, diets, and healthcare provisions, which help reduce the impact of unmeasured variables. However, there are limitations that need to be acknowledged. First, military personnel are a unique population and whether the results could be applied to the general population of young adults should be clarified. Second, the absence of information on coffee preparations, compositions and additives (e.g., sugar and milk), and the low daily coffee intake status is a major limitation. Although many covariates were controlled at baseline, a residual confounding may not be avoided, possibly leading to a bias. Third, the body composition of each participant was not assessed at baseline and during follow-up which may be used to understand the beneficial effects of coffee intake in advance.

## Conclusion

This cohort study provides substantial evidence to support a link between the guideline suggested moderate or more coffee intake in primary prevention and a reduction of future development of MetS among young adults after rigorous adjustments for potential confounders including moderate-intensity PA levels.

## Data availability statement

The raw data supporting the conclusions of this article will be made available by the authors, without undue reservation.

## Ethics statement

The studies involving humans were approved by the Institutional Review Board (IRB) of Mennonite Christian Hospital in Hualien City, Taiwan, with certificate number 16-05-008. The studies were conducted in accordance with the local legislation and institutional requirements. The participants provided their written informed consent to participate in this study.

## Author contributions

K-ZT: Formal analysis, Investigation, Methodology, Resources, Software, Writing – original draft. W-CH: Investigation, Supervision, Validation, Visualization, Writing – review & editing. XS: Investigation, Supervision, Validation, Visualization, Writing – review & editing. CL: Investigation, Methodology, Supervision, Validation, Visualization, Writing – review & editing. G-ML: Conceptualization, Data curation, Funding acquisition, Investigation, Methodology, Project administration, Resources, Supervision, Validation, Visualization, Writing – original draft.
